# Improved Methods of Counter-Extension in Treatment of Fractures of the Inferior Extremities

**Published:** 1864-04

**Authors:** E. Andrews

**Affiliations:** Professor of Surgery in Chicago Medical College


ARTICLE XIV.

### IMPROVED METHODS OF COUNTER-EXTENSION IN TREATMENT OF FRACTURES OF THE INFERIOR EXTREMITIES.

BY E. ANDREWS, M.D., Professor of Surgery in Chicago Medical College.

DR. LATTA, of Goshen, Indiana, has devised and contributed to the Museum of Chicago Medical College a new fracture apparatus, which is deserving of favorable mention among the improvements of the day. This instrument is constructed entirely of steel and iron, and though composed of a considerable number of pieces, yet has the different parts so firmly constructed and attached together that there is little liability to loss of the pieces or derangement of action. The chief peculiarity consists in the mode of counter-extension, which is effected by using the sound thigh as the base of pressure. The extension is attached to the affected limb by adhesive straps. The extending rod is of steel, and passes between the two limbs up to near the perineum, where it bears a short cross-arm. This cross-arm carries at either end a piece of bivalve sheet iron armor to embrace each thigh. The one for the sound limb encloses it firmly, so that the taper of the thigh prevents the armor from slipping upward, and thus furnishes a secure counter-extension. The armor for the broken limb embraces it more loosely, simply keeping it in position. The lower end of the extending bar is cut into a screw and carries a movable cross-arm, to which is attached a spring balance for showing the number of pounds of extension force applied. A hook in the cross-arm receives the ends of the adhesive straps coming from the affected limb, and the extension is made by screwing the cross-arm thus attached downward. A variety of conveniences exist in the apparatus, the details of which cannot be described in this article. The same instrument can be applied both to adults and to children, as well as to fractures on either side, or in any part of the limb, between the hip and the ankle. It is found by experience that Dr. LATTA'S invention possesses the following advantages:—

1st. The counter-extension has its bearing upon so large a surface that it is entirely painless.

2d. The attachment of the counter-extension being below the pelvis, it leaves the body free, and allows the patient to relieve tediousness of his confinement by sitting up in bed whenever he chooses.

3d. It preserves to the fullest extent the admirable benefits of adhesive strap extension.

It has the slight disadvantage of, to a certain extent, imprisoning the sound limb, and also of being some what complex in structure, though simple in action. The whole apparatus is neatly put up in a box.

In this connection I may be excused for completing the discussion by recalling other improvements which have already been noticed in the pages of the EXAMINER. Dr. DODGE, of Janesville, Wis., exhibited, at the last meeting of the American Medical Association, an apparatus for fractures of the inferior extremity, with an improved mode of making counter-extension by means of adhesive straps. This consists of a long splint, extending from below the foot to six inches above the crest of the ilium. At the upper extremity of the splint arises a semicircular steel bow, which passes over the shoulder and terminates in a hook by the side of the neck. To this are attached four long adhesive straps, each two and a-half or three inches broad, two of which extend down the back and two down the front of the body, as far as the pelvis. Thus a secure attachment is obtained for counter-extension, which is so large as to be entirely painless. The extension is made by adhesive straps, applied in the usual manner, which pass down to the foot-board. The foot-board itself is borne by a cross-arm, and extended by a screw.

The advantages of this instrument are, its painless counter-extension, the elastic action of the steel bow, and the simplicity and lightness of the apparatus. There is a certain degree of disadvantage, however, in the fact that the counter-extension being made from above the shoulder, the whole body is drawn straight, like a string, and kept more rigidly in one posture than is quite desirable; but the cures produced by it are free from shortening.

I have referred in a former paper to the fact that some surgeons have returned to the old practice of producing counter-extension by means of the weight of the hips on the single inclined plane, and are thus enabled to dispense entirely with all special counter-extending apparatus. In constructing appliances for this purpose, I advise the following method:—Have a joiner make a simple fracture box, four feet and six inches in length, by twelve inches wide at one end, and six at the other. The wide end may be a little hollowed out to fit against the bulge of the nates. The sides should be six inches high, and extend from the small end two and a-half feet towards the opposite extremity; then insert a screw pully in the small extremity, and the instrument is finished. The fractured limb should be carefully placed in this box, on suitable cushions, and the foot end of the apparatus raised at an angle of twenty or thirty degrees. Adhesive straps should be attached to the leg in the ordinary manner, and a cord run from them over the pulley, supporting a weight of several pounds. A bag of sand is a convenient weight, but a brick will often be found just as good. Some surgeons are so in love with this mode that they do not even place any short splints upon the thigh, but rely purely upon the extension to keep the bony fragments in correct position. On the whole, there can scarcely be anything devised better than this for plain, ordinary cases, as the counter-extension, being merely the weight of the hips, is painless, and the body of the patient is comparatively free to make all necessary motions. The box, however, is a little clumsy and unwieldy, when the surgeon wishes to take it with him from place to place.

Since the invention of adhesive strap-extension, there has remained only one difficult problem to be solved in fractures of the femur, and that was how to make efficient counter-extension without pain. This desideratum is now supplied by at least three good methods, so that we may truly say that the treatment of this class of surgical injuries has very nearly reached perfection.

